# 3D-Printed Ceramic Bone Scaffolds with Variable Pore Architectures

**DOI:** 10.3390/ijms21186942

**Published:** 2020-09-22

**Authors:** Ho-Kyung Lim, Seok-Jin Hong, Sun-Ju Byeon, Sung-Min Chung, Sung-Woon On, Byoung-Eun Yang, Jong-Ho Lee, Soo-Hwan Byun

**Affiliations:** 1 Department of Oral and Maxillofacial Surgery, Korea University Guro Hospital, Seoul 08308, Korea; ungassi@naver.com; 2 Department of Otorhinolaryngology-Head & Neck Surgery, Dongtan Sacred Heart Hospital, Hallym University College of Medicine, Dongtan 18450, Korea; enthsj@hanmail.net; 3 Department of Pathology, Dongtan Sacred Heart Hospital, Hallym University College of Medicine, Dongtan 18450, Korea; byeon.sunju@welovedoctor.com; 4 R&D Center, Genoss, Suwon 16229, Korea; soodentist@naver.com; 5 Department of Oral and Maxillofacial Surgery, Dentistry, Dongtan Sacred Heart Hospital, Hallym University College of Medicine, Dongtan 18450, Korea; drummer0908@daum.net; 6 Graduate School of Clinical Dentistry, Hallym University, Chuncheon 24252, Korea; face@hallym.or.kr; 7 Department of Oral and Maxillofacial Surgery, Dentistry, Sacred Heart Hospital, Hallym University College of Medicine, Anyang 14068, Korea; 8 Department of Oral & Maxillofacial Surgery, School of Dentistry, Seoul National University, Seoul 03080, Korea; leejongh@snu.ac.kr

**Keywords:** hydroxyapatite, tricalcium phosphate, 3D printing, digital light processing, ceramic scaffold, pore architecture

## Abstract

This study evaluated the mechanical properties and bone regeneration ability of 3D-printed pure hydroxyapatite (HA)/tricalcium phosphate (TCP) pure ceramic scaffolds with variable pore architectures. A digital light processing (DLP) 3D printer was used to construct block-type scaffolds containing only HA and TCP after the polymer binder was completely removed by heat treatment. The compressive strength and porosity of the blocks with various structures were measured; scaffolds with different pore sizes were implanted in rabbit calvarial models. The animals were observed for eight weeks, and six animals were euthanized in the fourth and eighth weeks. Then, the specimens were evaluated using radiological and histological analyses. Larger scaffold pore sizes resulted in enhanced bone formation after four weeks (*p* < 0.05). However, in the eighth week, a correlation between pore size and bone formation was not observed (*p* > 0.05). The findings showed that various pore architectures of HA/TCP scaffolds can be achieved using DLP 3D printing, which can be a valuable tool for optimizing bone-scaffold properties for specific clinical treatments. As the pore size only influenced bone regeneration in the initial stage, further studies are required for pore-size optimization to balance the initial bone regeneration and mechanical strength of the scaffold.

## 1. Introduction

The use of bone grafts on bony defects caused by tumors, trauma, or aging is an established and standardized procedure which has been clinically successful over a long period of time [1,2,3]. Many reports have been published on successful bone healing after autogenous, allogeneic, xenogeneic, and alloplastic bone grafting [4,5,6]. Furthermore, successful outcomes have been reported for bone grafts performed using growth factors such as bone morphogenic protein (BMP) [7,8,9]. Most commercial graft materials are currently composed of powder, which can be used to fill bone defects without any bony gaps [10]. However, powder-type bone-graft materials take a long time to graft and may detach from the wound site, making it difficult to graft areas where physical support is required [5,11]. Therefore, the development of a new block-type bone-graft material is required to overcome the limitations of powder materials. However, commercial graft blocks need to be in close contact with the defects and need to be trimmed to fit the wound shape, which is one disadvantage when screw removal is required [12].

Three-dimensional printers have facilitated the development of customizable block-type bone-graft materials [13,14]. To date, polymers have mainly been used as 3D-printed bone substitutes [14]. However, the high molecular weights of certain polymers result in irregular pore sizes, which negatively affect cell-adhesion behavior of the scaffold [15,16]. Polymers also have lower bone-conduction abilities than those of the calcium-phosphate-based materials [17]. Hydroxyapatite (HA)/tricalcium phosphate (TCP) has excellent biocompatibility, biodegradation, osteoconductivity, and osteoinductivity [18,19,20,21]. Therefore, HA/TCP is considered a suitable ceramic material for manufacturing bone grafts using 3D printing. However, 3D printing and posttreatment processes that can produce pure ceramic materials have not been sufficiently studied compared to polymer-based 3D printing. Therefore, it is difficult to develop a technology for achieving pure ceramic 3D-printed bone-regeneration materials.

Typically, 3D-printed scaffolds are fabricated using fused deposition modeling (FDM) 3D printing methods. Recently, digital light processing (DLP) 3D printing has been used to produce bone scaffolds from a mixture of osteoceramic powder and polymeric binder [22,23]. Most previous studies did not attempt to completely remove the polymer binder due to concerns regarding the mechanical stability of the scaffold. However, a recent study showed that it is possible to completely remove the polymer from DLP 3D-printed parts using a heat-treatment step, and the structure did not collapse [24].

Appropriate pore features could enhance the biomechanical properties of the scaffold, enabling good mechanical interlocking between the grafted scaffold and adjacent bone, thereby enhancing bone regeneration in the 3D-printed pore spaces of the scaffolds [25]. Pore features, such as the size, architecture, porosity, and connectivity, influence the cellular activities and bone regeneration [26,27,28,29,30]. Poor control of the pore features using FDM 3D printing has made it difficult to develop bioceramic scaffolds for bone regeneration. With high accuracy and sensitivity, the DLP 3D-printing technique is an advanced method of fabricating biocompatible bioceramic scaffolds [31]. However, the ideal pore architecture for improving bone regeneration in 3D-printed scaffolds has not yet been studied sufficiently [22,32]. The novel contribution of this study is the elucidation of the effect of pore size on the bone generation performance of pure ceramic scaffolds produced using DLP 3D printing.

In this study, a DLP 3D printer was used to fabricate block-type scaffolds of pure HA/TCP after complete removal of the polymer binder. Using a rabbit calvarial model, this study aimed to evaluate the mechanical and bone-regeneration properties of 3D-printed scaffolds with variable pore design/size. We hypothesized that the pore sizes of the graft specimens would have significant effects on bone regeneration.

## 2. Results

### 2.1. Compressive Strength

Analysis of the 3D-printed HA/TCP scaffold blocks showed that the compressive strength of the sample with the 1.0-mm cubic pores (6.20 ± 0.8142 MPa) was higher than that of the diamond pores (2.80–3.60 MPa). In the case of the 0.9-mm diamond pores, when a frame was present, the compressive strength (3.60 ± 0.9119 MPa) was higher than that of the sample with no frame (2.80 ± 0.8246 MPa). The sample type with cubic pores and no frame was used for the remaining studies because it had the highest compressive strength of those tested.

### 2.2. Cytotoxicity and Clinical Findings

After 24 h and 48 h, the grades of the cells using the negative and positive control eluates were zero and four, respectively. The eluate of the control showed no change. The grade of the cells in the HA/TCP specimens was zero at both 24 h and 48 h, thereby confirming no cytotoxicity [24].

The animals did not show any specific clinical signs of death during the observational period. Additionally, no abnormal symptoms, infections, or inflammation were visually observed on the operated sites. In the fourth and eighth weeks, no abnormalities in the skull shape or unusual lesions were observed at the time of euthanasia.

### 2.3. Bone Formation and Pore Size

**Figure 1:**
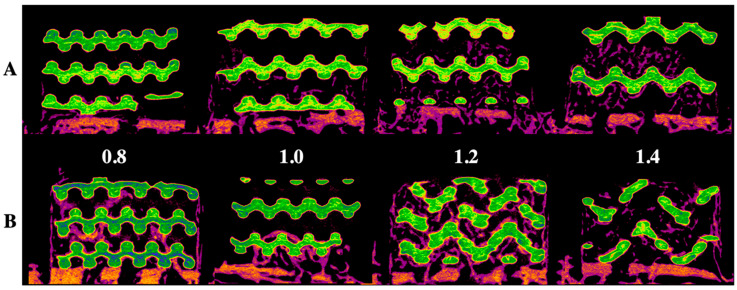
Radiological evaluation of the 3D-printed HA/TCP scaffold with pore sizes of 0.8, 1.0, 1.2, and 1.4 mm: (**A**) after four weeks, (**B**) after eight weeks; green color: HA/TCP scaffold and purple color: newly generated bone.

**Figure 2:**
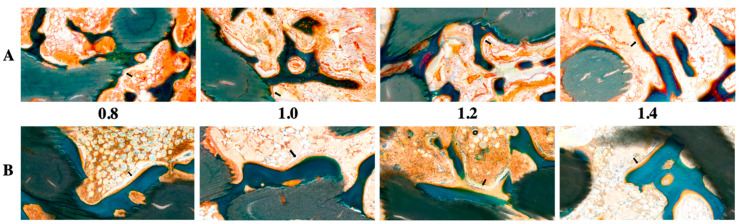
Histological evaluation of 3D-printed HA/TCP scaffolds with pore sizes of 0.8, 1.0, 1.2, and 1.4 mm: (**A**) after four weeks (black arrow: osteoblast) and (**B**) after eight weeks (black arrow: osteoblast); green color: HA/TCP scaffold.

Figure 1 and Figure 2 show radiological and histological images, respectively, used to determine the percentage of new bone formation at 4 and 8 weeks (as listed in Table 1). Both analyses showed that larger pore sizes promoted bone formation after four weeks (*p* < 0.05). Less bone formation was observed for 1.0-mm and 0.8-mm pore sizes than for 1.4-mm and 1.2-mm pore sizes (*p* < 0.05). At four weeks, no significant difference and no significant similarity between bone formations were observed in samples with pore sizes of 0.8 mm and 0.8 mm and of 1.2 mm and 1.4 mm, respectively. In the eighth week, bone formation increased by approximately 2.5 times compared to the fourth week, and a relationship between pore size and bone regeneration was not observed (*p* > 0.05).

The histological images (Figure 2) obtained using GT staining showed that nonspecific inflammatory reactions associated with foreign bodies, such as necrosis and granuloma formation, did not occur in any of the groups. Most of the groups had granulation tissues inside the graft in the fourth week, but in the eighth week, the granulation tissues were reduced and changed to hematopoietic cells.

## 3. Discussion

The mechanical strength of the 3D-printed bone is less than that of the natural human bone. Nevertheless, researchers have endeavored to increase the mechanical strength and bone regeneration ability of the 3D-printed bone for clinical use. This study compared the compressive strengths of different 3D-printed bone designs. A cubic pore design showed better compressive strength than that of a diamond pore design with the same pore size. Therefore, the cubic pore design was used for all further analyses as its high compressive strength would facilitate bone regeneration procedures. Among the various types of 3D-printing systems for biomedical purposes, most previous studies have focused on the FDM 3D-printing system to fabricate bone materials [33,34]. FDM typically combines HA or TCP powders with polymers through a nozzle. Polymers such as polycaprolactone, polylactic acid, and poly-DL-lactic acid are used, as they can melt at temperatures high enough to dissolve the TCP and to maintain the shape and structure of the 3D-printed ceramic materials. Owing to the presence of the polymer, these HA/TCP composites have fairly poor biocompatibility and limited capability to form new bone [35,36]. Additionally, owing to the large nozzle diameter (approximately 0.4–0.5 mm) in FDM-type 3D printers, it is difficult to print bone-graft materials with high-resolution features.

Very few studies on HA/TCP scaffolds have been conducted using a DLP 3D-printing system [37]. Unlike an FDM-type 3D printer, the DLP system used in this study reduces the processing time by simultaneously irradiating the entire cross section and produces a biomaterial with a higher resolution than those of the other systems [38]. DLP technology uses a digital projector as a light source, which significantly reduces printing time and enables highly accurate production [39]. The DLP system uses a ceramic slurry to produce ceramic scaffolds in a layer-by-layer polymerization process [40,41]. As HA and TCP are not responsive to light, they need to be mixed with photoactive polymers that harden when exposed to UV light [42]. Subsequent heat treatment is used to completely remove the polymers and to prepare a highly biocompatible scaffold containing only osteoceramic materials. The complete removal of the photopolymer depends on the heating rate, temperature, and time [43]. The specific methods for polymer removal, HA/TCP slurry fabrication, and 3D printing are trade secrets in the 3D-printing market of bioceramics.

In this study, the safety and quality of the 3D-printed bone were both verified through a biological safety assessment. For all pore sizes, the complete removal of the polymer was confirmed by SEM examination and cytotoxicity tests, indicating that complete polymer removal could be achieved using an appropriate heat-treatment process. This study also proved the biocompatibility and bone-regeneration properties of the 3D-printed bone substitutes, which have the same composition as that of conventional HA/TCP alloplastic bone while providing additional structural stability.

In this study, bone formation was observed for scaffolds with different pore sizes over an 8-week observation period. The ideal pore size of ceramic scaffolds for enhancing bone regeneration has been discussed in previous studies [44,45], and different ideal values have been proposed. Chang et al. reported that more bone was formed in the scaffolds with larger pore sizes (300 and 500 μm) [46], similar to the findings of Gauthier et al. [47]. Vassilis et al. showed that ceramic scaffolds with pore sizes >300 μm enhanced bone regeneration and vascularization [48]. They argued that hypoxic conditions caused by small pores induce osteochondral formation, while with the aid of good vascularization, large pores lead to direct osteogenesis. Our results are consistent with the findings of these previous studies. It was thought that larger pore sizes enhance uniform bone formation inside the scaffold during the initial healing period. However, given enough time, it seems that similar bone formation occurs regardless of pore size. In contrast, a previous study found that a pore size of 100 μm gave optimal bone formation among β-TCP scaffolds with three pore sizes (100, 250, and 400 μm) in a calvarial defect model [49]. Although the optimal pore size was not determined in this study, we will examine this issue in the near future. In addition, the mechanical properties should be optimized through further studies.

In addition, the mechanical performance of the ceramic scaffold is critical during bone formation as it affects the degree of scaffold degradation, new bone formation, and degree of osteointegration into the surrounding bone. Consistent with most ceramic materials, smaller pore sizes result in better mechanical performance [49].

## 4. Materials and Methods

### 4.1. Fabrication of 3D-Printed HA/TCP Scaffold Blocks

**Figure 3:**
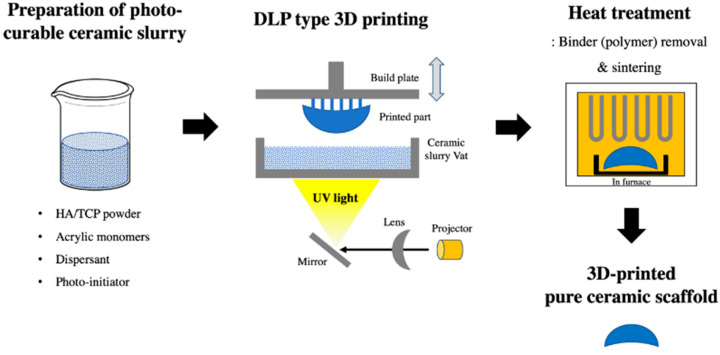
Digital light processing (DLP) 3D-printing process of the pure hydroxyapatite (HA)/tricalcium phosphate (TCP) scaffold.

A 3D-printed scaffold substitute prepared using a DLP 3D printer (Cubicon Lux, Cubicon^®^, Sungnam, Korea) was used as a synthetic bone graft material. This 3D printer has a resolution of 100 μm and can print layers with a thickness layer of 20–100 μm. The scaffold was designed with four variable pore architectures (0.8, 1.0, 1.2, or 1.4 mm). The bone graft designs (Φ6 mm × 4 mm) were converted into a stereolithography file that was used by the 3D printer to form the samples in a layer-by-layer manner. The precursor material was a photoreactive ceramic-resin composite (slurry) composed of HA/TCP (6:4 ratio; Dentium^®^, Suwon, Korea), a dispersant, acrylic monomers, and a photo-initiator (Phenylbis phosphine oxide; Sigma-Aldrich^®^, St. Louis, MO, USA). The photoreactive ceramic–resin composite was prepared by blending 64 wt.% ceramic powder with a proprietary resin formulation. The slurry was placed in the vat of the 3D printer, which has a transparent bottom through which the ultraviolet light is projected to polymerize the slurry and to form the sample, which is attached to a build plate that slowly moves upward during printing. After the printing was completed, the scaffold was carefully removed from the build plate and then thoroughly cleaned with copious amounts of distilled water to remove excess liquid slurry. Then, the polymer was completely removed using a heat-treatment step, where the scaffolds were sintered at 1250 °C for 10 h in an electrically heated chamber furnace (Carbolite, Ubstadt-Weiher, Germany) in ambient air [50]. The polymer residue was removed during sintering by pyrolysis, and the pure ceramic scaffold was obtained (Figure 3).

**Figure 4:**
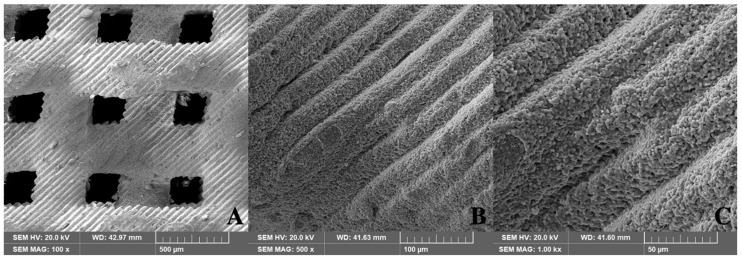
Structure of 3D-printed HA/TCP scaffolds (SEM, 0.8 mm pore): (**A**) 100×, (**B**) 500×, and (**C**) 1000×.

The pore structure of the 3D-printed scaffold was thoroughly evaluated using scanning electron microscopy (SEM). Figure 4 shows the SEM images of pure HA/TCP scaffolds without a residual polymer.

### 4.2. Compression Tests

The compressive strength of the specimens was evaluated to determine the relationship between the mechanical properties and pore structure. Three specimen designs were used: 0.9-mm diamond pore, 0.9-mm diamond pore with a frame, and 1.0-mm cubic pore (Figure S1). Block-type specimens were prepared for each pore design with a cross-sectional area of 1 cm^2^ and a height of 2 mm. ASTM D695 “Standard Test Method for Compressive Properties of Rigid Plastic” was used in this study. After specimen placement, the rod of the universal testing machine (H50K-T UTM^®^, Tinius Olsen Corp., Surrey, UK) was lowered at a rate of 0.5 ± 0.1 mm/min, and the load applied to the sample was measured. The extracted load data were calculated by dividing the strain by the cross-sectional area of the specimen. A log-linear plot of the stress (N/cm^2^) as a function of distance (μm) was plotted. Compressive stress values were derived from the graph by locating the intersection of linear fits of two regions, corresponding to an initial rapid increase in stress and a region where the stress gradually increased.

### 4.3. Cytotoxic Tests

The in vitro cytotoxicity test standard (ISO 10993-12) was used to evaluate the specimen cytotoxicity (3 specimens per pore size). The 3D-printed HA/TCP specimens were eluted for 24 h at 37 °C in 20 mL of elution solvent. A minimal essential medium (MEM; 500 mL) was prepared by adding 50 mL of fetal bovine serum (Gibco^®^, Thermo Fisher Scientific, Green Island, NY, USA) and 10 mL of penicillin-streptomycin solution (Welgene^®^, Gyeongsan-si, Korea). High-density polyethylene and natural rubber were used as negative and positive control materials, respectively. The control solutions were eluted under the same conditions. Mouse fibroblasts (ATCC CCL 1, clone 929 of strain L, Korean Research Institute of Bioscience and Biotechnology, Daejeon, Korea) were injected into flasks containing the MEM with antibiotics and fetal bovine serum, and the flasks were maintained in an incubator at 37 °C. The culture solution was changed three times a week.

Cells attained 80% confluence after one day of culture in a 6-well plate. After complete removal of cell culture media, 2 mL of the elution solvents from the negative control, positive control, and experimental specimen were added to and cultured in 6-well plates. After culturing for 24 h and 48 h, cell growth and lysis levels were measured using a microscope. The condition of the cells was evaluated from the grade of the cells. When the grade of the negative control eluate was zero and that of positive control eluate was higher than 4, the test was considered reliable. If the grade of the experimental specimen was lower than 2, no cytotoxicity occurred [24].

### 4.4. Animal Experiments

**Figure 5:**
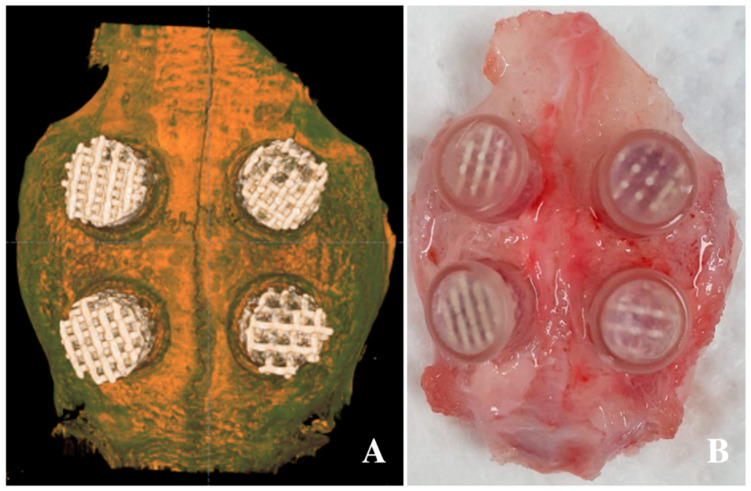
Application of 3D-printed HA/TCP scaffolds in the defect of the calvarium: (**A**) radiographic image after application of the scaffolds and (**B**) clinical photo after scaffold application.

Twelve seven-week-old male New Zealand white rabbits (2.6 ± 0.3 kg in weight) were used in this study. General anesthesia using xylazine HCl (Rompun^®^, 10 mg/kg, Bayer Korea, Seoul, Korea) and a combination of tiletamine and zolazepam (Zoletil^®^, 50 mg/kg, Virbac Korea, Seoul, Korea) were administered intramuscularly. Betadine was used to prepare shaved forehead surgical sites; an additional injection of 1:100,000 epinephrine was administered subcutaneously for hemostasis, and an incision was made on the forehead. Four defects were designed on each calvarial bone, and a circular border was made with an 8-mm-diameter drill. Additionally, nine holes of 1-mm diameter were created within the circle for blood supply and bone regeneration [51]. Customized polycarbonate tubes (Φ8 mm × 5 mm) were fitted and fixed into the circular border, and each specimen, with a different pore size (0.8, 1.0, 1.2, or 1.4 mm) and a diamond pore design, was inserted into the tube. Then, the tube was covered with a lid to prevent the sample from falling off (Figure 5) [52]. Layer-to-layer continuous suturing was applied using 4–0 Vicryl^®^ (Johnson & Johnson, Brunswick, NJ, USA). Diclofenac (5 mg/kg, IM, Sinil, Seoul, Korea) and gentamicin sulfate (5 mg/kg, IM, Sinil, Seoul, Korea) were injected postoperatively for three days as a postoperative analgesic and an antibiotic, respectively. The animals were monitored weekly for wound dehiscence, inflammation, infection, and general health until they were euthanized. Six animals were chosen for euthanasia four and eight weeks after the surgery. After being anesthetized using xylazine HCl and a combination of tiletamine and zolazepam, the animals were euthanized by administering KCl to the marginal ear vein. After euthanasia, the surgical site was dissected and the skull was removed and fixed with 10% formalin solution. This experiment was approved by the Genoss Laboratory Animal Ethics Committee based on the law on laboratory animals (animal laboratory approval No: GEN-IACUC-1908-01, August 2019). All animal experiments complied with the ARRIVE (Animal Research: Reporting of In Vivo Experiments) guidelines and were performed in accordance with the UK Animals (Scientific Procedures) Act, 1986 and associated guidelines, EU Directive 2010/63/EU for animal experiments, or the National Institutes of Health guide for the care and use of laboratory animals.

### 4.5. Micro-CT Imaging

The extracted skulls were fixed to the center of a micro-computed tomography (μCT) machine (SkyScan1173^®^, Ver. 1.6, Bruker-CT, Kontich, Belgium), and μCT imaging was performed. The imaging conditions were as follows: 130 kVp tube voltage, 60 μA tube current, 1-mm aluminum filter, 500 ms exposure time, and 0.3° rotation angle. The number of pixels was 2240 × 2240, and the pixel size was 13.85 μm. A total of 800 high-resolution images were obtained. NRecon (Ver 1.7.0.4, Bruker-CT, Kontich, Belgium) was used for cross-sectional reconfiguration, whereas Dataviewer (Ver. 1.5.1.2, Bruker-CT, Kontich, Belgium) and Ct-VOX (Ver. 1.14.4.1, Bruker-CT, Kontich, Belgium) software were used for 3D construction. The intensity value range of 55–255 (0: black; 255: white) was used to identify the bone volume in the region of interest for radiological analysis. The newly formed bone volume was calculated using these programs, as follows: percent bone volume (%) = bone volume/tissue volume.

### 4.6. Histological Analysis

After leaving the extracted specimens for one week in formalin, they were rinsed with flowing water for 9 h and then cut for processing. The specimens were dehydrated with increasing concentrations of EtOH in the order of 70%, 95%, and 100% EtOH. Then, the tissue sample was infiltrated with ethyl alcohol and Technovit 7200 Resin (Heraeus KULZER, Hanau, Germany) in the form of a mixed solution with increasing resin ratios. Finally, penetration was performed by vacuuming the stock solution of Technovit 7200 resin for two days. The resin was cured using a UV embedding system (KULZER EXAKT 520^®^, Heraeus KULZER, Hanau, Germany). Then, the resin block was cut in half using an EXAKT Diamond cutter (KULZER EXAKT 300^®^, Heraeus KULZER, Hanau, Germany) to produce a cross section around the material and bone tissue. The thickness of the initial section was 300 ± 50 μm, which was polished to a thickness of 40 ± 5 μm using an EXAKT grinding machine (KULZER EXAKT 400CS^®^, Heraeus KULZER, Hanau, Germany). Tissue slides were produced and stained using a Goldner’s trichrome (GT). The images of tissue slides were obtained with an optical microscope (OLYMPUS BX50^®^, Olympus Optical CO. Tokyo, Japan) equipped with a charge-coupled device camera. Measurements were recorded with objective lenses at magnifications of ×1.25 and ×4, whereas ×10 and ×20 objective lenses were used for high resolutions. Subsequently, the area of the new bone was calculated using analysis software (Image-Pro Plus^®^, Media Cybernetics, Rockville, MD, USA) as follows: new bone (%) = area of new bone/total area of defect × 100.

### 4.7. Statistical Analysis

The Kruskal–Wallis test was used for statistical interpretation and to analyze the differences in bone regeneration among the four groups. Comparisons between groups were calculated with Mann–Whitney U tests, and results with *p*-values <0.05 were considered as statistically significant. Statistical analysis was performed using SPSS 20 for Windows (SPSS Inc, Chicago, IL, USA).

## 5. Conclusions

Block-type bone scaffolds were manufactured using a DLP 3D printer from a ceramic-polymer slurry. We demonstrated that it was possible to completely remove the polymer by a subsequent heat treatment step to produce pure HA/TCP scaffolds. In this research, pure HA/TCP scaffolds with pore architectures clinically suitable for bone regeneration could be produced using DLP 3D printing. The pore size of the scaffold influenced bone regeneration in the initial stage, although the total amounts of bone regeneration in the final stage were similar for all the pore sizes. Although larger pore sizes enhance bone regeneration in the initial stage, such scaffolds can have lower mechanical strength. Therefore, further studies are required to determine the ideal pore size for balancing the trade-off between initial bone regeneration and mechanical strength.

## Figures and Tables

**Table 1:** New bone formation of 3D-printed HA/TCP scaffolds with variable pore sizes.

Analysis	Weeks	0.8	1.0	1.2	1.4	*p*
Radiological	4 weeks	4.166 ± 0.302	4.921 ± 0.274	5.903 ± 0.274	5.750 ± 0.327	<0.05 *
%	8 weeks	8.684 ± 1.340	7.709 ± 1.442	9.607 ± 1.314	8.802 ± 1.354	>0.05
Histological	4 weeks	3.798 ± 0.278	4.087 ± 0.267	5.256 ± 0.201	5.257 ± 0.152	<0.05 *
%	8 weeks	10.245 ± 0.444	10.745 ± 0.334	9.493 ± 1.407	8.292 ± 2.716	>0.05

Average ± standard deviation; * statistical significance at *p* < 0.05.

## Supplementary Materials

Supplementary Materials can be found at https://www.mdpi.com/1422-0067/21/18/6942/s1. Figure S1: Specimen designs for compression test.
