# Supplementary figures and images for: 3D-Printed Ceramic Bone Scaffolds with Variable Pore Architectures
Source: Int J Mol Sci. 2020 Sep 22;21(18):6942. doi: 10.3390/ijms21186942 (PMC7555666; doi:10.3390/ijms21186942)

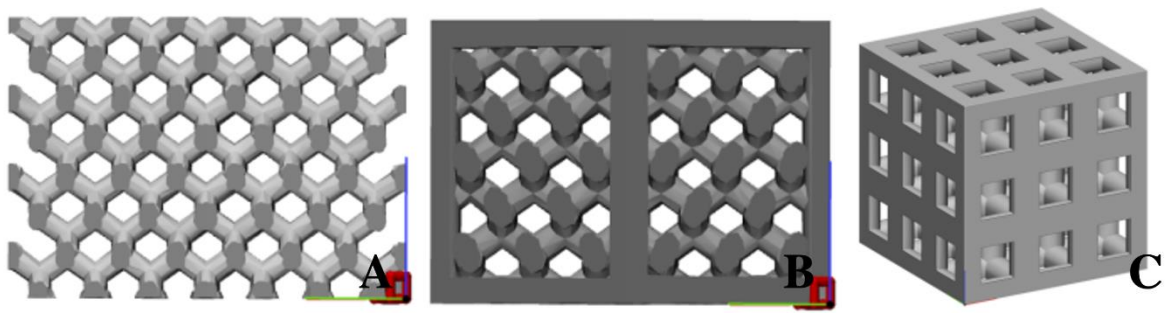

**Figure S1.** Specimen designs for compression test.

Supplement: Supplementary file 1 [file ijms-21-06942-s001.pdf]
